# Mapping the experiences of people with achalasia from initial symptoms to long‐term management

**DOI:** 10.1111/hex.13160

**Published:** 2020-11-19

**Authors:** Melika Kalantari, Amelia Hollywood, Rosemary Lim, Majid Hashemi

**Affiliations:** ^1^ School of Pharmacy University of Reading Reading UK; ^2^ University College London Hospitals NHS Foundation Trust London UK

**Keywords:** Achalasia, chronic condition, diet, patient experiences, process map, rare condition, self‐management, stress

## Abstract

**Background:**

Achalasia is a rare motility disorder affecting the oesophagus, which is associated with a range of symptoms and different treatment strategies. Currently, little is known about people's experiences with achalasia and its management. This study aimed to understand the experiences of people living with achalasia, from the initial onset of symptoms to long‐term management.

**Method:**

This qualitative study explored the journey of people living with achalasia and outlined the care pathway using a process map. Ten female and five male participants living with achalasia (age range: 40‐73) took part, and all aspects of their diagnosis, treatment and management were discussed. A process map showing people's experiences by separating the management of their condition into a series of steps was developed to present the pathway in the participants’ journey. The analysis involved discussing the process map within the research team.

**Results:**

The process map comprised of 10 steps, which occurred before and after diagnosis. The developed map indicates that most participants managed their on‐going symptoms through stress management techniques and dietary changes. Key issues that participants highlighted about their journey managing achalasia were misdiagnosis, delay in diagnosis and lack of support in the long‐term management of achalasia.

**Conclusions:**

This research was a novel study exploring patients’ experiences and management of achalasia and mapping their journey. Two distinct phases to their journeys were identified: before and after diagnosis. Areas highlighted by this study can provide a basis for future research, in particular behaviour change to support the long‐term management of achalasia.

## 1 INTRODUCTION

Achalasia is a rare motility disorder affecting the oesophagus. This condition can start at any time of life but is more common in middle‐aged or older adults.^1^ It is equally prevalent in males and females, with an overall incidence of 1.63 cases per 100 000 people.^2^, ^3^ The underlying causes of achalasia are unknown.^1^ Characteristic features, such as a non‐relaxing sphincter, weak or absent oesophageal peristalsis and simultaneous or poorly coordinated contraction, lead to an outflow obstruction at the level of the lower oesophageal sphincter (LOS). The presence of these features leads to difficulty in swallowing, particularly with liquids before solids, as well as a variety of other associated symptoms. Patients can present with several years’ history of progressive symptoms, or acutely with a range of symptoms such as complete dysphagia, regurgitation and progressive weight loss.^1^ The symptom onset is typically insidious and usually progress over many years, and it has a substantial impact on quality of life.^2^ The diagnosis of achalasia can be confirmed by different diagnostic tests such as endoscopy, barium swallow and manometry.^1^ Despite it being a crippling condition, about 20‐50% of cases are initially misdiagnosed, with patients given an alternative diagnosis such as gastro‐oesophageal reflux disease or hiatus hernia.^4^ Treatments for achalasia are often delayed due to a lack of diagnosis, and even the most effective treatments usually do not achieve a cure.^2^ Therefore, in a large proportion of patients, the initial treatment is either delayed or inappropriate and ineffective.^5^ Moreover, as the cause of achalasia is unknown, treatment has focused on alleviating the symptoms and their consequences. There are different treatment options available for achalasia such as medications, Botox injections, balloon dilatation, surgical interventions and non‐medical interventions such as behavioural changes. It is therefore critical that people with achalasia learn to self‐manage their symptoms (to some degree).

Behavioural and lifestyle adjustments are required for successful management of chronic conditions.^6^ Chronic disease management is typically conducted by the patient in their day‐to‐day life; however, their interaction with health‐care providers is a critical intersection for information exchange, decision making and motivation.^7^ Chronically ill patients have to manage their daily living under different financial and social constraints, and their associated symptoms can complicate the most routine activities of daily living.^7^ According to a study carried out by Clark et al,^8^ three separate categories of activities need to be addressed in order to successfully self‐manage a chronic condition.^8^ First, people with chronic conditions need to have an adequate knowledge about their condition and its treatment to make informed decisions. Second, they need to perform activities in order to manage their condition by making changes to their lifestyle, including changing their dietary habits. Third, they need to apply skills to maintain adequate psychosocial functioning, which includes working, maintaining a good family life and cultivating social relationships.^8^ These self‐management activities aim to reduce the impact of chronic conditions on daily life.

It is challenging to live with any chronic condition, in particular a rare disease such as achalasia. The general public often shows sympathy and understanding when someone lives with a visible or well‐known disease; however, living with a rare or less visible disease generates different challenges for individuals and those who interact with them.^9^ Knowledge, coping strategies and problem‐solving skills are factors that enhance adjustment and adaptation to a long‐term chronic condition.^9^ A better understanding of their condition helps people to cope with the anxiety and uncertainty, while lack of knowledge leads to a sense of powerlessness.^10^

Health‐care professionals who do not have the knowledge to diagnose a rare disease may mistakenly label their patients as ‘psychological cases’ rather than persons having an individualized physical illness.^9^ This is demonstrated in results from the study of another rare condition, scleroderma, in which nurses with very little knowledge about this rare condition stigmatized individuals by ignoring the signs and symptoms of the condition and labelling them as ‘untrustworthy’.^9^ Stigma can produce a sense of fear and rejection among people with such a health condition.^9^ This is further evident historically where, for example, people with epilepsy were stigmatized as dangerous or violent due to a lack of knowledge amongst the general public.^6^

Exploring people's experiences can be a valuable way of providing first‐hand information about their care pathways. There is often a lack of appropriate health services, effective treatment options and skilled health professionals for rare conditions.^11^ Patients can be the best source of information to give appropriate suggestions relating to their experiences and areas of their care which need improvement.^11^ Rare diseases have a significant psychological and emotional impact on patients and their families, which is often followed by a lack of appropriate community and peer support.^11^ Research has explored different treatment options for achalasia in terms of their effectiveness, but the focus has not been on the patient's experience and in particular the individual's journey along their pathway. The current study thus aimed to map the experiences of people living with achalasia from diagnosis through to long‐term management and to suggest where further input is needed from health‐care professionals.

## 2 METHOD

### 2.1 Design

An exploratory qualitative study using a process map was conducted. Quantitative studies such as surveys were not appropriate as this was exploratory work. The data provided in the mapping sessions can be used to improve patient pathways.^12^ This study involved in‐depth, semi‐structured mapping sessions to explore and understand the care pathways for people living with achalasia and the long‐term management of this condition. This study used a phenomenological approach to describe the lived experiences of people with achalasia ^13^ and allow the researcher to understand and gain rich details on an unknown area.

### 2.2 Methodology

This study involved three mapping sessions, which were conducted in London, United Kingdom. The research materials, including the topic guide and draft map, were prepared by the research team before each session. A process map was produced in each session, and information was added cumulatively. The process map produced in the final session included all the steps involved in the participant journey, which was discussed in the three sessions from the first onset of the symptoms through to the on‐going management of achalasia. The final process map was discussed and refined within the research team. A favourable ethical opinion was granted through the University of Reading Research Ethics Committee (UREC‐13/38).

### 2.3 Paradigm underpinning the methodology

Constructivism was the approach used to interpret the data that were collected in the sessions. This approach considers that there could be multiple interpretations of an event, shaped by the researcher's historical or social perspective in comparison with positivism, which adheres to the factual knowledge gained through observation and measurements.^14^ This research was a qualitative study to understand a particular phenomenon; therefore, data were interpreted based on the researcher's expertise.^14^ In this approach, it is acknowledged that the researcher interprets the data based on their own understanding and knowledge developed through their own experiences.^15^ The researchers facilitated the sessions using prompts to elicit the participants’ experiences. Through the analysis, the researchers constructed meaning from the data based upon their previous knowledge and research to produce the final process map.

### 2.4 Recruitment

Participants were recruited through the Achalasia Support Group affiliated with Achalasia Action, an independent charity supporting people living with achalasia in the United Kingdom (UK). This is a national group, with over 400 members, based in London and run by members of the public living with achalasia. People usually find and join this group through searching on the Internet and word of mouth. Convenience sampling was the strategy used to recruit participants based on their willingness and availability to take part. The inclusion criteria for this study were as follows: anyone living in the UK, aged 18 years or over, with a confirmed diagnosis of achalasia, who consented to take part, was able to attend a session and spoke English. The researcher emailed the recruitment materials, which included an information sheet and consent form, to the administrator of the support group who distributed these through the support group mailing list inviting members to participate. Participants were asked to contact the researcher (MK) with their interest in taking part in a mapping session.

### 2.5 Data collection

Before the first session, a basic draft of a process map was produced by the researcher based on preliminary research,^16^ which involved interviewing individuals living with achalasia about their experiences. Based on the preliminary research, the draft process map consisted of three main stages, which were pre‐diagnosis, diagnosis and after diagnosis to facilitate discussion in the mapping sessions. The draft process map was used to provide structure to the discussion.

### 2.6 Process mapping sessions

Participants in the current study were allocated to one of the three mapping sessions based on their availability. Mapping sessions were held over a month with a maximum of eight people in each session. The aim of the study, the structure of the session and the topics for discussion were stated at the beginning of each session. The ground rules were explicitly stated and included confidentiality, raising concerns during sessions and the option to withdraw from participation. After their consent had been obtained at the beginning of each session, participants were given a brief demographic questionnaire asking their sex, age, living status (living alone or co‐habiting) and the type of medical interventions they had received for achalasia. The sessions lasted around 90‐120 minutes and were audio‐recorded for accuracy check purposes.

In each session, participants were asked to talk through their journey from the first onset of their symptoms through to the on‐going management. The researchers provided prompts to participants and recorded their experiences. Two researchers were present at each session to ensure all the points that were raised were documented. At the end of each session, the researchers checked all points raised were documented and then participants were provided with stickers to add to the discussion board to highlight the most challenging steps in their journey. The collected data in the mapping sessions produced a process map, and the recordings were used for accuracy check purposes only. Participant quotes were not transcribed and included in the process maps as the main aim of this study was to explore the overall care pathways and steps involved in people's journey living with achalasia. In this methodology, data are collected in real time and a process map is created in the mapping sessions. Two female researchers facilitated each session. Session 1 was run by two pharmacist researchers, and the following sessions were run by a pharmacist and a health psychologist.

### 2.7 Data analysis

The collected data were discussed within the research team after each session. The content with similar ideas or concepts was grouped and then assigned headings corresponding to key steps in people's journeys, resulting in a more detailed and informative draft map for the second session. The process map and its contents were edited and analysed through listening repeatedly to the recordings. Recordings were reviewed to check the accuracy of the process map, and they were listened to repeatedly to identify the steps and the iterative process of people's journeys. Findings were analysed by the research team to address the aim of the research. The analysis involved discussing the findings based on the number of steps in their journey, number of times the patients had been sent from one health‐care professional to another, time taken in and between each step, steps that added no value for the participants and areas where problems were raised for them.^17^ Through this analysis, process map was developed and validated by the participants in the final session where participants were asked to confirm whether the process map was an accurate representation of their journey. Data saturation was achieved in the final session. Data saturation is reached when there is enough information to replicate the study and when the ability to gain additional new information has been attained.^18^ The final process map is the end product of the analysis, and it gives an outline of the findings. The researchers created the final process map iteratively by analysing the constructs in detail to develop a map that most accurately represents the participants’ journey. The final developed process map presents the discrete steps in a participant's journey in the order in which they occurred.

## 3 RESULTS

### 3.1 Sample

This study included 15 participants (5 males and 10 females) with achalasia. The participants ranged in age from 40 to 73 years (mean 56). One person contacted the researcher and then decided not to take part due to the group setting involved in the mapping session. Most participants were white British, and living with a partner, family or friend (co‐habiting) (see Table 1 for demographic details of the participants). Disease management reported by participants ranged from a year to 20 years. Two participants participated in session 1, eight in session 2 and five in session 3.

**Table 1:** Participants’ demographics

Participant's demographics	All participants (n = 15)	
Sex	Male	n = 5 (33%)
	Female	n = 10 (67%)
Age (years)	Mean (SD)	56.2 (13.3)
	Range	40‐73
Ethnicity	White	n = 15 (100%)
Living status	Living alone	n = 4 (27%)
	Co‐habiting	n = 11 (73%)
Employment status	Full time	n = 5 (33%)
	Part time	n = 3 (20%)
	Retired	n = 5 (33%)
	Not working	n = 1 (7%)
	Not stated	n = 1 (7%)
Medical treatment	Multiple treatments (medication and/or surgery)	n = 4 (27%)
	Single treatment	n = 8 (53%)
	No treatment	n = 2 (13%)
	Not stated	n = 1 (7%)

### 3.2 Overview of findings

**Figure 1:**
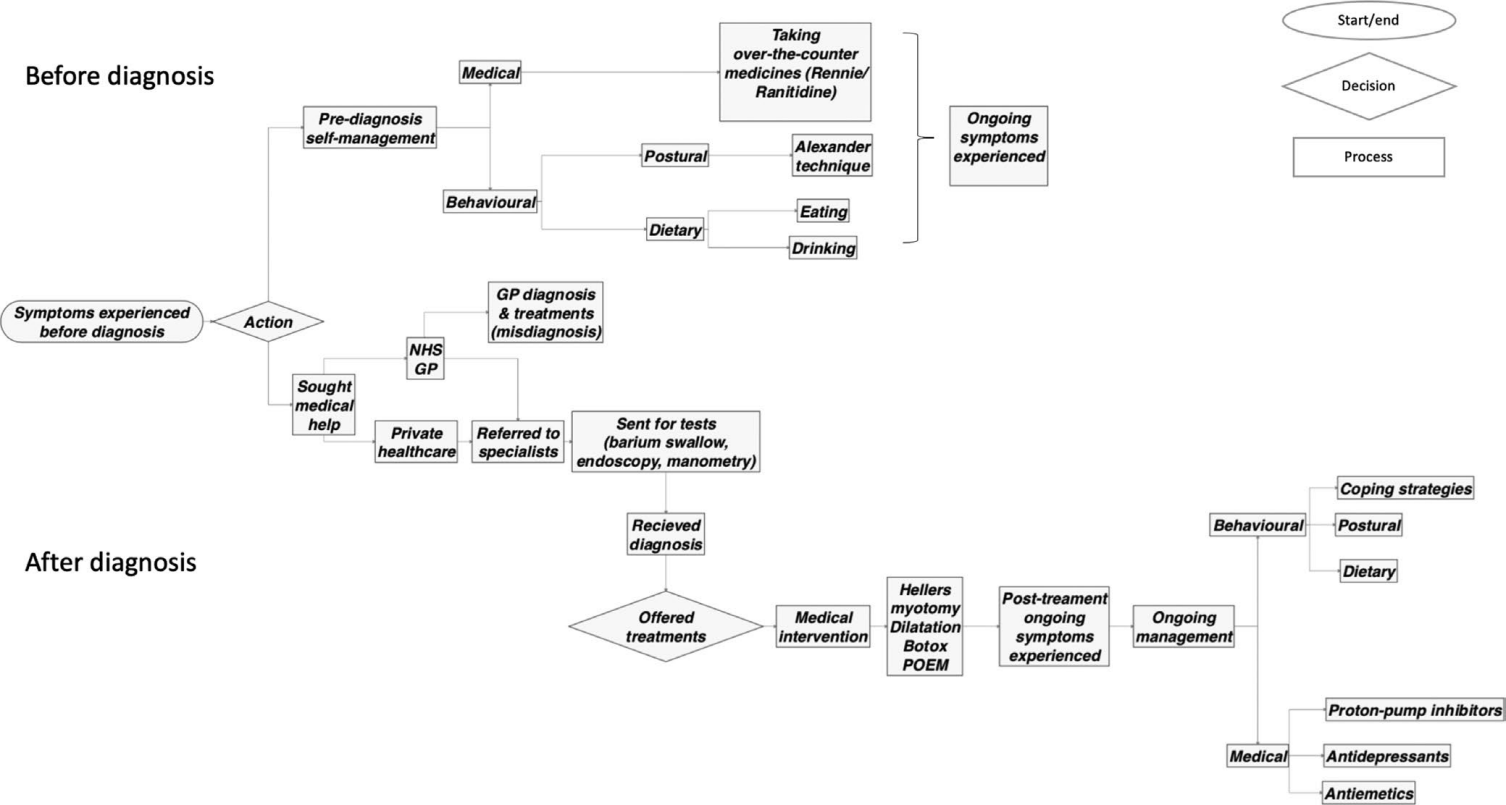
Process map of participants’ journey

Participants provided rich insights into their journey living with achalasia. Figure 1 shows the key steps in the participants’ journeys. The results show that there were two main phases (before and after diagnosis) with 10 steps in total. These steps were as follows: 1. symptoms; 2. management; 3. seeking medical help; 4. diagnosis based on the symptoms experienced; 5. return to GP; 6. sent for tests; 7. offered treatments; 8. post‐treatment complications or symptoms; 9. on‐going management; and 10. general impacts. These steps were the headings used in the mapping sessions and provide the structure for this results section. The process map created after the three sessions was the end product, and the rich details led to developing the process map. Similarities were identified amongst participants’ responses in the onset of the symptoms, the tests they received for diagnosis and the treatments offered. Key issues that participants highlighted included the initial misdiagnosis, the delay in receiving the diagnosis and the lack of additional support throughout their journey. Figure 1 is the final version of the process map, illustrating a summary of the participants’ journey with achalasia from the first signs and symptoms through to on‐going management.

### 3.3 Before diagnosis

Apart from a few participants who visited a private health‐care professional, the majority of the participants visited their National Health Service (NHS) general practitioner (GP). Due to the similarity of the symptoms of achalasia to other conditions, participants were often given treatment for another condition, which was unsuccessful. They became stuck in a loop where they had to repeatedly visit their NHS GP until being sent for an appropriate test and referred to a specialist for diagnosis. Participants who visited a private health‐care professional reported obtaining a diagnosis quicker as they were sent for the appropriate diagnostic tests immediately.

#### 3.3.1 Symptoms experienced by participants before diagnosis

The majority of participants experienced a range of symptoms initially, such as chest pain, regurgitation, difficulty swallowing and heartburn. Some also reported experiencing recurrent burping, breathlessness and choking.

#### 3.3.2 Pre‐diagnosis management

After initially experiencing symptoms, people with achalasia took two different routes, with some seeking medical help to find out the underlying cause of their symptoms and the rest taking actions to self‐manage their symptoms. Some people tried to manage their symptoms by buying over‐the‐counter medicines, including indigestion remedies such as Rennie or Ranitidine. Some addressed them indirectly simply by adjusting their diet unconsciously and eating what they found easier to swallow. One participant reported ‘googling’ her symptoms to find the underlying cause.

#### 3.3.3 Seeking medical help

After attempting to self‐manage their condition, participants reported seeking medical help and visiting their GP when there was no improvement in their symptoms and the symptoms persisted. The majority of participants visited their GP through the NHS to obtain a diagnosis. Other participants, however, sought medical help through their private medical insurance. Those who went to a private health‐care professional reported getting a diagnosis earlier than those who accessed a GP via the NHS.

#### 3.3.4 Misdiagnosis and treatment

Symptoms experienced by people with achalasia are very similar to other conditions; therefore, it was often misdiagnosed. Aortic aneurysm, acid reflux, angina and hiatus hernia were a few common conditions that people were diagnosed with at their many visits to the GP. They were getting treatments for these conditions but had to go back to their GP repeatedly due to on‐going issues such as not being able to eat or drink, experiencing painful spasms and losing weight.

#### 3.3.5 Return to GP

Participants revisited their GP many times before being referred to a specialist and sent for appropriate tests to get a diagnosis. One of the participants reported having to demonstrate how she struggled to swallow water before being sent for tests. After multiple visits to their GP, participants were eventually referred to address their symptoms. The initial referral varied, with participants, referred to an oncologist, ENT (ear, nose and throat) specialist or gastroenterologist or sent directly for tests.

#### 3.3.6 Sent for tests

The common tests which almost all participants were sent in varying order included barium swallow, endoscopy, manometry and computerized tomography (CT) scan. After undergoing several tests, participants reported receiving a diagnosis and being offered treatments to choose from.

### 3.4 After diagnosis

Once participants obtained a diagnosis, they were offered a medical intervention, such as endoscopic Botox injection, endoscopic balloon dilatation or laparoscopic Heller's myotomy. Participants either chose to undergo a procedure or manage their condition with non‐medical interventions (such as dietary changes). Once participants had an understanding of the condition, they attempted to self‐manage. Participants had to change their dietary habits, such as the timing of their meals or choosing foods that were easier to swallow, and also tried changing their posture at night by sleeping in a more upright position. At this time, participants had a few options, including choosing one or more treatments, managing their condition by behavioural changes, accessing information about their condition, and joining an online support group to get more help. They also reported issues such as struggling to find accurate information on the management of their symptoms and insufficient support from the health‐care team after diagnosis.

#### 3.4.1 Offered treatment

Only one participant reported a significant delay in communication receiving a diagnosis from her GP after the diagnostic tests. Many reported that health‐care professionals offered only endoscopic or surgical treatments, instead of any lifestyle or behavioural changes that could have benefited them in the on‐going management of their condition. Participants also emphasized a lack of support and communication from health‐care professionals at this stage. Most of the participants were not satisfied with the level of information provided about the treatment options from the clinicians, or with the aftercare they received from the nurses at the hospital they visited. Only one participant reported managing their condition with no further medical or surgical treatment.

#### 3.4.2 Post‐treatment complications or symptoms

Participants, in general, complained about the lack of follow‐up and monitoring after treatment. They believed they were more susceptible to illnesses due to inability to consume nutritious food. Almost all participants reported that the treatments did not resolve their symptoms completely and that they were still experiencing symptoms such as heartburn, indigestion and pain. Some participants experienced repeated treatments, whereas others continued to self‐manage their symptoms. At this stage, self‐management included taking over‐the‐counter products such as indigestion remedies (eg Gaviscon), exercise (eg tai chi and yoga) and dietary changes.

#### 3.4.3 On‐going management

Participants sought to manage their condition through self‐management, using behavioural and medical strategies. Some participants reported changing behaviours such as their diet and lifestyle, while others were taking therapeutic medicines such as anti‐depressants and cannabis oil to cope with their condition. The majority of participants used a combination of strategies to reduce the recurrence of symptoms. Almost all reported educating themselves by searching online and communicating with other members of the online support group. The on‐going management of their condition was reported as one of the most crucial points in the journey, as support was required in the long‐term.

#### 3.4.4 General impacts on day‐to‐day living

Participants referred to achalasia as a hidden disability, an unseen illness that has a significant impact on social life and employability. Achalasia significantly affected their lives, and they reported feeling anxious, stressed, exhausted and depressed, which meant they could not perform their daily tasks as they would have liked.

## 4 DISCUSSION

This is the first study to the authors’ knowledge that explored people's experiences of living with achalasia using a process map. People living with achalasia experience a complicated journey from seeking a diagnosis through to post‐diagnosis. The complexity of their journey is mainly due to the ambiguity of symptom presentation, lack of knowledge from doctors and the rarity and chronic nature of the condition. There is no one way to manage the symptoms of achalasia; therefore, people living with this condition need to manage their symptoms by trying different self‐management strategies through trial and error. The key findings of this study were the steps involved in people's journey living with achalasia and the areas in their journey which participants found difficult. These key areas were the delayed diagnosis and the difficulties they experience in the on‐going symptoms management, specifically the lack of support from health‐care professionals involved in their care. These were the areas where an intervention could be implemented to help them in the on‐going long‐term management of this condition. This study also highlighted the lag in the initial stages where people with achalasia are misdiagnosed, and they make multiple visits to the doctors to receive a diagnosis. The key message of this novel study is despite different successful evidence‐based medical interventions people living with achalasia continue to experience symptoms, such as reflux and pain, and have to self‐manage their condition. This is a potential area that an intervention could be developed and implemented to support the on‐going long‐term management of achalasia.

Before receiving a diagnosis, people had to deal with uncertainty and anxiety. Furthermore, as they did not know what they were living with, the lack of knowledge about their condition led to a feeling of helplessness. Most were stuck in a loop of obtaining a misdiagnosis, with treatment directed towards the symptoms based on the erroneous diagnosis. Many discussed how not knowing about the condition and its underlying causes and treatments made them anxious. Continued uncertainty about a condition can affect the quality of life, for example by increasing levels of depression and emotional distress.^19^ Mishel et al defined uncertainty as to the inability to determine the meaning of illness‐related events which arises when there is a lack of information to understand one's illness, treatment and side‐effects.^20^ They also defined uncertainty as to when individuals are unable to use health‐care professionals or related support to obtain knowledge which they need in order to develop a strategy for coping with an unfamiliar situation.^20^ Our empirical for achalasia patients are consistent Mishel et al theoretical account of uncertainty. In chronic conditions, the experience of uncertainty spreads to wider life issues. Familiar routines are disrupted and perceptions of the order of life are altered.^21^

Self‐management was a common approach in all the steps involved in the journey of patients with achalasia from before diagnosis to after treatment. Participants described a range of self‐management strategies, including medical and behavioural adjustments, such as dietary changes, to cope with their condition, even though some had undergone successful medical or surgical treatments. Achalasia patients need to self‐manage as do people with other chronic conditions as shown in previous research. Corbin and Strauss suggest three self‐management tasks for chronic illnesses: behavioural management (adjusting lifestyle), emotional management (managing anger, fear or frustration) and medical management (taking medication).^22^ They recommend to accomplish these tasks, and people with chronic conditions ought to apply core self‐management skills, such as problem‐solving, decision making, resource utilization, taking action and partnership with health‐care providers.^21^ Participants in our study also reported using these core skills to a varying extent through their journey. In a study on HIV, eight main categories of self‐management strategies emerged: (a) take medication or treatment; (b) modify activity; (c) alter food in the diet; (d) seek help; (e) wait; (f) substance use; (g) manage thoughts or attitude; and (h) alter physical environment.^23^ These self‐management strategies were also described by the participants in our study suggesting some core management approaches across disease types.

After diagnosis, participants were offered different treatment options. Their treatment choices were based mainly on the health‐care professional's advice and recommendations, along with, to some extent, on the minimal information available to participants regarding treatments. The International Society for Disease of the Oesophagus developed a guidelines in 2018 intending to offer clinicians and patients an up‐to‐date framework for making informed decisions on the management of this rare condition.^24^ Fifty‐one experts from 11 countries and three representatives from patient support associations participated in preparing these guidelines. Even though the consensus agreement score of the available treatments was more than 80% and most of the available treatments were considered successful, the majority of patients who participated in developing the guidelines were still experiencing symptoms after undergoing treatment.^24^ Achalasia, like many other chronic conditions, requires on‐going treatment and continuous self‐management as it has no cure and the available treatments serve only to alleviate the symptoms. Patients attempt to use a variety of personal, social and health‐care resources to lessen the treatment burden.^25^ The results of our study are consistent with other evidence which links social support, optimism and spirituality to better well‐being in chronic conditions.^25^ Therefore, although the available treatments are reported to be more than 80% successful, they are not 100% effective in all patients. The process map produced in this study maps out the journey of patients and gives an insight to health‐care professionals to see what patients go through from the first onset of their symptoms to the on‐going management of achalasia. The produced process map can also be a useful decision aid for health‐care professionals. Moreover, it can assist health‐care professionals for better shared decision making.

### 4.1 Strengths and limitations

The mapping process in this study allowed the researchers to receive first‐hand and in‐depth insight into participants’ experiences with this rare chronic condition. Process mapping is a useful method, which has not previously been used to understand patient's experiences living with achalasia by separating the management of the condition into a series of steps allowing the researcher to see the sequence of steps involved in their care pathway. It is an interactive approach that collects ideas from people who are involved in the same process. The data collected for producing the process map can be used to redesign patient pathways and improve the quality or efficiency of management of a disease such as achalasia and alter the focus of care towards activities that are most valued by patients. The nature of this method let participants discuss their journey within a group setting and collect ideas from a group of people, which resulted in the end product of the process map that interviews alone would not allow. Participants were asked to talk about their journey from before diagnosis through to the on‐going management, with further details added in each session. People in the last session confirmed that the developed map was an accurate representation of their journey. The current study explored a rare condition using qualitative methods to gain an insight into the steps involved in people's journey. The process map provides structure and enables evaluation of the steps involved in their journey.

There were several limitations to this research. Although the sample size was small, it was appropriate for the method used^17^; future research may use a quantitative approach such as questionnaires to access the experiences of people with achalasia outside the UK in a larger scale. Participants were recruited through a support group, which provides the opportunity for its members to share experiences and tips with others; their on‐going disease management may, therefore differ from that of people who are not part of such a support group. People were asked to attend and engage in an in‐depth mapping session and share sensitive information about a significant period in their lives, and this could affect how they reported certain events. The voluntary nature of participation meant that people who took part may have had a different experience to others who are not willing to participate in research. The results presented in this paper are solely based on what was described in the sessions and the interpretation of the researchers, and its findings should, therefore, be viewed from that perspective.

With regard to credibility, the multidisciplinary background of the research team enables us to explore different perspectives while interpreting the data. We used convenience sampling in order to make sure that the selected participants were representative of the variety of views and background to ensure the transferability of results.^26^ The findings from this study are representative of a white, mainly female, adult sample who live in the UK and were able to speak English. Theoretical saturation was achieved through the three mapping sessions; therefore, data collected was stopped as no new information was added to the process map.

### 4.2 Implications for practice and research

The findings from this study have implications for both clinical practice and future research. The process map produced in this study maps out the journey of patients and gives an insight to health‐care professionals to understand what patients go through from the first onset of their symptoms to the on‐going management of achalasia. It also provides new insights into the patient's journey from the patient's perspective, which could be used to improve the doctor‐patient relationship. Understanding the patient's journey from their perspective could enable and encourage health‐care professionals to tailor the care of individuals based on their journey and can also be a useful decision aid for health‐care professionals. Moreover, it can assist health‐care professionals for better shared decision making. The results also identified that participants felt that they did not receive enough information and support throughout their journey and for the long‐term management of achalasia. Misdiagnosis by the NHS GPs was also very common. These findings highlight the need to co‐develop patient resources to find out what resources, interventions and support patients think they would benefit from, along with further GP training. The results from this study also identified the key time points and the steps where people feel they need additional support. Stress was a key component, which triggered patients’ symptoms at different phases, and was highlighted as a potential area for future research. A stress management programme could be developed as a tool in enabling people to deal with the anxiety and stress associated with this condition.

### 4.3 Future research

Future studies should design or co‐design an intervention with people with achalasia to initiate a behaviour change so it can help people to self‐manage their condition better, and further studies should include health‐care professionals as co‐producers of research knowledge (such as doing research collaboratively as opposed to doing research on people with achalasia) to underpin further work on developing interventions to enable better disease management.

## 5 CONCLUSION

This study mapped the journey of people with achalasia. The study highlighted the issues people faced at each stage in their journey and identified the areas that need addressing to help people cope with their condition, including interventions to improve patient care. The process map also highlighted the importance of self‐management of chronic conditions. People who participated in this study may have undergone different medical treatments, but all of them were still experiencing symptoms that required them to adopt different self‐management strategies to carry out their normal lives.

## CONFLICT OF INTEREST

The authors declare no potential conflicts of interest with respect to the research, authorship and/or publication of this article.

## Data Availability

The data that support the findings of this study are available from the corresponding author upon reasonable request.
